# From the Field to the Lab: Best Practices for Field Preservation of Bat Specimens for Molecular Analyses

**DOI:** 10.1371/journal.pone.0118994

**Published:** 2015-03-23

**Authors:** Angelique Corthals, Alynn Martin, Omar M. Warsi, Megan Woller-Skar, Winston Lancaster, Amy Russell, Liliana M. Dávalos

**Affiliations:** 1 Department of Sciences, John Jay College of Criminal Justice (CUNY), New York, New York, United States of America; 2 Department of Pathology, Stony Brook University School of Medicine, Stony Brook, New York, United States of America; 3 Department of Biology, Grand Valley State University, Allendale, Michigan, United States of America; 4 Department of Ecology and Evolution, Stony Brook University, Stony Brook, New York, United States of America; 5 Department of Biological Sciences, California State University, Sacramento, California, United States of America; 6 Consortium for Inter-Disciplinary Environmental Research, School of Marine and Atmospheric Sciences, Stony Brook, New York, United States of America; University of Illinois at Urbana-Champaign, UNITED STATES

## Abstract

Studies in molecular ecology depend on field-collected samples for genetic information, and the tissue sampled and preservation conditions strongly affect the quality of the DNA obtained. DNA yields from different tissue types have seldom been compared, and the relative performance of storage media has never been directly tested, even though these media may influence DNA degradation under field conditions. We analyzed DNA yield from buccal swabs and wing punches harvested from live bats using nucleic acid quantification as well as quantitative PCR for a single-copy nuclear locus. We also compared DNA yields from wing tissue preserved in three media: ethanol, NaCl-saturated dimethyl sulfoxide (DMSO), and silica desiccant. Wing punches yielded more total DNA than did buccal swabs, and wing tissues preserved in silica beads yielded significantly more total and nuclear DNA than those preserved in DMSO or ethanol. These results show that tissue type and preservation media strongly influence the quantity of DNA obtained from non-lethal genetic samples, and based on these effects we provide recommendations for field collection of tissues for genetic analyses.

## INTRODUCTION

Collecting samples in the field for molecular studies can be stressful or even lethal for the organism of interest. Ecologists must often sample tissues in a way that both maximizes nucleic acid yields and minimizes stress on the sampled individual. The choices of tissue type and tissue preservation protocols are often based on convenience and historical practices instead of optimizing for the highest yield. In this paper, we aim to evaluate alternative, affordable, and efficient field collection protocols that do not require euthanizing the animal for downstream molecular analyses. We focus on bats (Mammalia: Chiroptera) based on both our expertise and the burgeoning literature on chiropteran population genetics and phylogenetics [1–5].

Traditionally, the most common mammalian tissue collected in the field has been the liver, because it is both easy to identify and very large [6–15]. If the goal is to retrieve genomic DNA for sequencing, however, the liver is ill suited for preservation as it has several distinct disadvantages for molecular analysis. First, it requires the euthanasia of the animal being sampled. Second, because of its high enzymatic activity, the liver is also the first organ to go through autolysis after death [16]. Third, livers express high levels of stress- and inflammation-related proteins at the moment of euthanasia, as transient hypoxia increases hypoxanthine and xanthine in liver cells [17]. This may bias transcriptomic or proteomic studies, and contaminates genomic DNA extractions with plentiful RNA transcripts that may interfere with sequence capture methods [18]. The second most common tissue type, muscle, is more resistant to autolysis, but also requires the euthanasia of the animal sampled and thus may contribute to population declines [8,14,19].

Nonlethal sampling of bats usually involves obtaining patagial biopsy samples [20–22] or feces [23]. While Worthington et al. [21] advised storing patagial samples in NaCl-saturated dimethyl sulfoxide (DMSO), ethanol and silica beads are also commonly used as storage media [15,24,25]. These three media are affordable and relatively easy to transport, and have been the preferred method of nonlethal bat tissue preservation in the field. Each medium, however, generates challenges in preserving even robust molecules such as genomic DNA [12,13]. Other agents, such as lysis buffer, or Qiagen’s RNA*later* and AllProtect are used less often. The Qiagen products are relatively expensive (e.g., RNA*later* costs $315 US/250 ml), and lysis buffer over-lyses tissues when left for a long period of time at room temperature or in field conditions [26,27]. Ideally, field-collected samples should be preserved in liquid nitrogen to preserve the best molecular profile, including fragile molecules [28,29]. This preservation method is rarely used in the field, though, as liquid nitrogen is difficult to transport and attracts negative attention at customs and airports, and refilling tanks is often difficult or impossible in remote locations [8,30–32].

In this paper, we assess several affordable methods of sampling and preserving mammalian tissues without euthanizing individuals. We sampled bat wing punches and mouth swabs aiming to uncover the best type of tissue for downstream genomic DNA analyses. The collection of buccal cells using a mouth swab has been extensively used for rabies surveillance [33], and is being introduced here to collect bat epithelial cells. Three common media (ethanol, DMSO, and silica beads) were used to assess tissue preservation methods for wing punches. Genomic DNA was extracted from each sample type and for each medium used. A comparison of DNA yields, using both spectrophotometric nucleic acid quantification and quantitative PCR (qPCR) of a single-copy nuclear gene, was conducted between sample types (wing punch vs. buccal swabs) and between each preservation medium for wing punches (ethanol, DMSO and silica beads). The purpose of both experiments was to evaluate which sample type and which medium produced the best results.

## RESULTS

**Fig 1:**
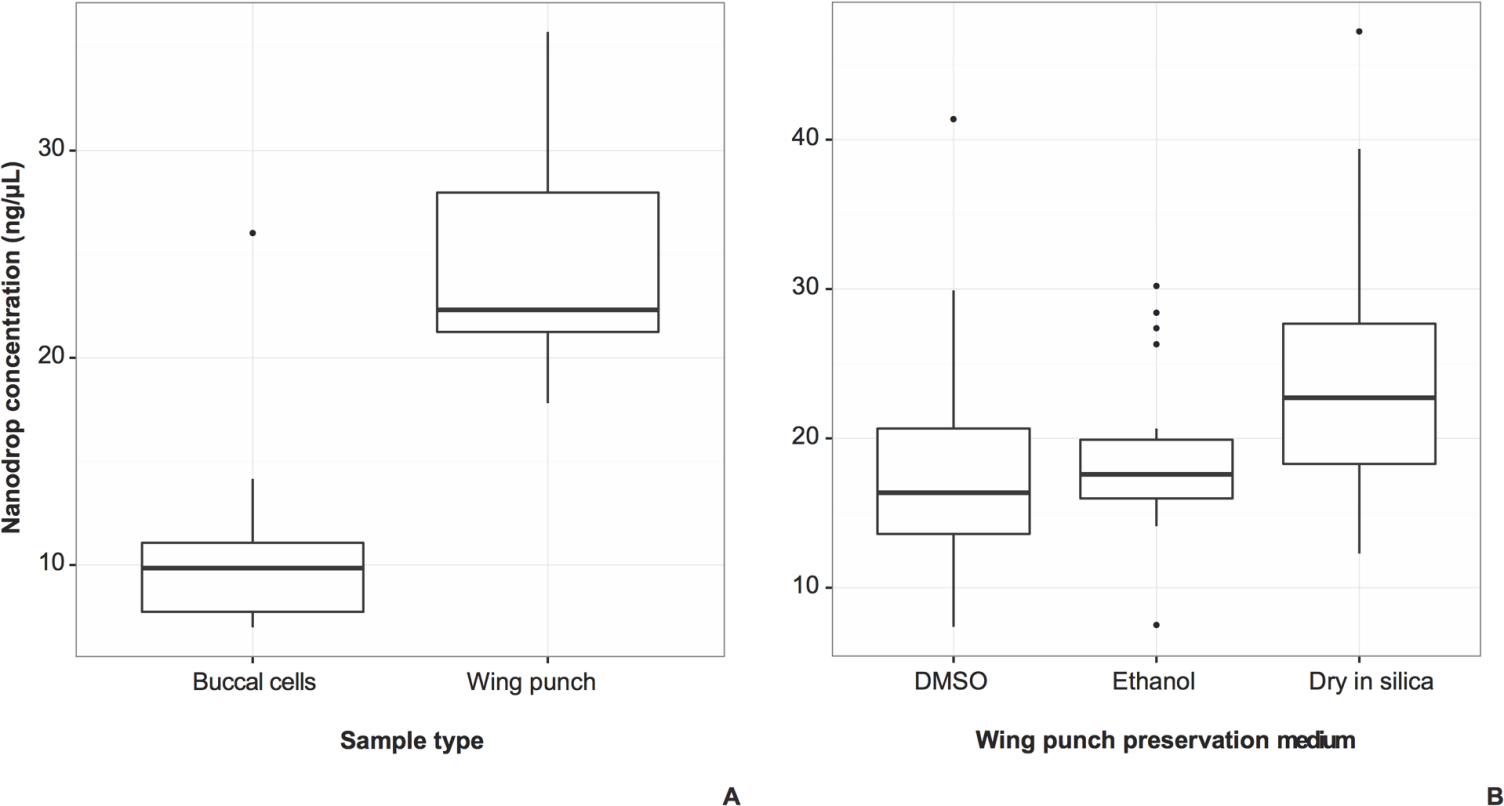
DNA concentration measured using nanodrop spectrophotometer. A. Comparison between sample types obtained from the same individuals (*t*_(22)_ = 6.55, *P* = 1.254e-06). B. Comparison between preservation treatments for wing punch samples obtained from the same individuals (individual effect *F*_(20, 36)_ = 5.250, *MSE* = 121.8, *P* = 8.33e-06; treatment preservation effect *F*_(2, 36)_ = 6.978, *MSE* = 161.9, *P* = 0.00275).

Optical density estimated through spectrophotometry was used to measure nucleic acid quantity and purity, which comprises nucleic acid quality and protein contamination. The 260/280 optical density ratio is a measure of the purity of nucleic acids, with measures above 1.80 indicating minimal protein and chemical contamination [34]. The 260/280 ratio of all extractions was in all cases ≥1.86, indicating that nucleic acids were not contaminated by proteins or other chemicals. Comparisons of total nucleic acid concentration from the sample type experiment showed that wing punches yielded more nucleic acids than buccal cells (Fig. 1A). Comparisons from the preservation medium experiment showed that more nucleic acids were recovered from wing punches preserved dry in silica than from DMSO- or ethanol-preserved wing tissue (Fig. 1B).

**Fig 2:**
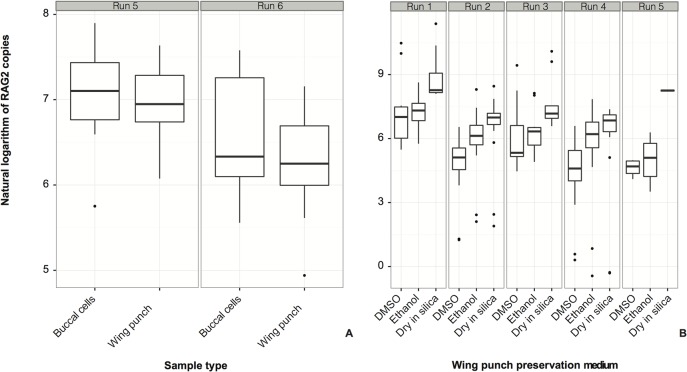
Number of copies of single-copy nuclear gene obtained through quantitative PCR (qPCR) amplifications from different sample types and methods of preservation. Results are presented sorted by qPCR run. The models that best fit both datasets accounted for group-level effects of runs and individuals sampled (Table 1). A. Comparison between sample types obtained from the same individuals (no significant effect of sample type was found, see Table 1). B. Comparison between preservation treatments for wing punch samples obtained from the same individuals (ethanol and dry preservation were significantly better than DMSO preservation, see Tables 1 and 2).

In contrast to the total nucleic acids recovered, target DNA quantity, as assessed by the number of copies of the nuclear gene amplified from buccal cells and wing punches, did not differ significantly between tissue types (Fig. 2A), and there was great variability between runs and individuals sampled (Table 1, S1 Fig.). The best-fit model included group-level coefficients for both qPCR runs and individual bats, but the effect of the sample type was not significant (Table 1). In the preservation medium experiment, wing punches preserved dry in silica in the field consistently yielded more copies of the nuclear gene than punches preserved in ethanol, which in turn produced more copies than punches preserved in DMSO (Table 2, Fig. 2B, S1B Fig.). As with the sample type experiment, the best-fit model accounted for both qPCR runs and individual bats and, in sharp contrast, preservation medium had a significant effect on DNA copies detected (Table 1). Table 2 provides an estimate of the effects of individual preservation methods, showing that silica beads preserved more copies than ethanol, and both preserved more copies than DMSO (for every copy preserved in DMSO, ~2.4 copies were preserved in ethanol, and ~5.7 in silica).

**Table 1:** Models of the natural logarithm of nuclear gene copies detected through qPCR as a function of the sample type (*N*_*observations*_ = 90, *N*_*individual bats*_ = 12, buccal swab brush or wing punch.

**Model**	**Group-level intercepts**	**DF**	**logLik**	**AIC**	**LRT**	***P*-value**
**No predictor**	qPCR run	3	−77.76	161.53	—	—
**Sample type**	qPCR run	4	−76.55	161.10	2.426	0.12
**No predictor**	Individual	3	−82.01	170.03	—	—
**Sample type**	Individual	4	−81.40	170.79	1.230	0.27
**No predictor**	qPCR run and individual	4	−69.94	147.87	—	—
**Sample type**	qPCR run and individual	5	−68.98	147.95	1.916	0.17
**No predictor**	qPCR run	3	−438.88	883.76	—	—
**Preservation medium**	qPCR run	5	−417.07	844.13	43.624	3.4e-10
**No predictor**	Individual	3	−359.94	725.87	—	—
**Preservation medium**	Individual	5	−298.09	606.19	123.68	0.00
**No predictor**	qPCR run and individual	4	−354.09	716.86	—	—
**Preservation medium**	qPCR run and individual	6	−282.32	576.64	145.53	0.00

This includes individual WCL025, not shown in S1A Fig. because it only had a wing punch), or the preservation medium for wing punches (*N*_*observations*_ = 228, *N*_*individual bats*_ = 21, silica beads, ethanol, or DMSO. This includes individual ALR074, not shown in S1B Fig. because it only had a wing punch preserved in ethanol). No-predictor models serve as null hypotheses of no effect of the sample type or preservation medium on the number of *rag2* copies detected using qPCR. Group-level intercepts of qPCR run and/or individual bat sampled serve to capture effects of unmeasured variability from subtle differences in qPCR starting conditions and bat behavior. Significance was measured using a likelihood ratio test of the model with predictors to its null. Models with different sample types or preservation media as predictors can be compared to one another using the Akaike Information Criterion (AIC). The model with the lowest AIC is the best fit to the data. DF = model degrees of freedom, logLik = log-likelihood of model fit to the data, and LRT = likelihood ratio test statistic.

**Table 2:** Estimated coefficients of the effect of wing punch preservation methods on the natural logarithm of gene copies detected after accounting for differences between qPCR runs and among individual bats.

**Medium**	**Coefficient**	**Std. error**
**Dry in silica**	6.94	0.39
**Ethanol**	6.08	0.39
**DMSO**	5.20	0.39

**Fig 3:**
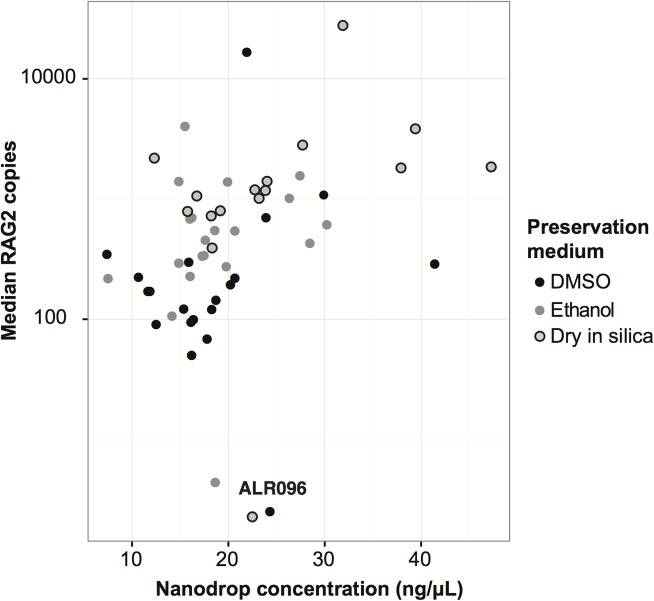
Relationship between the median number of copies of the single-copy nuclear gene obtained from repeated quantitative PCR reactions and concentration of whole DNA (wing punches in three preservation treatments). The correlation was significant whether including (*r* = 0.32, *p* = 0.018), or excluding the outlier measurements from sample individual ALR096 (*r* = 0.47, *p* = 0.00034).

The number of nuclear gene copies and concentration of total cellular nucleic acids in extracts were significantly associated in wing punches in the preservation medium experiment (Fig. 3). In contrast, nucleic acids and nuclear gene copies from matched buccal cells and silica-preserved wing punches in the sample type experiment were not significantly associated (S2 Fig.).

## DISCUSSION

Buccal swabs acquired in the sample type experiment required more collector skill and were less bat-friendly than initially anticipated. Though larger bats (e.g., *Brachyphylla*, ~35 g) could handle the swabbing with brushes unhurt, smaller bats (e.g., *Pteronotus quadridens*, ~5 g) did not fare well. The epidermis of the palate of smaller bats would quickly tear, causing discomfort and sometimes bleeding unless extreme care was applied. The samples themselves were also more prone to inconsistency during collection compared with the wing punches. While a wing punch is easily replicable by using a biopsy punch tool, collecting buccal cells depends on the time the collector is able to handle the bat, as well as the sensitivity of the bat to the swab brush. Though a time of 60 seconds was assigned to the collection of each buccal sample, not all bats handled were able to sustain 60 seconds of brushing without bleeding.

The buccal cells obtained by swabbing, though easy to transport, produced low nucleic acid yields (Fig. 1A), even as they generated similar numbers of copies of target nuclear DNA as wing punches (Fig. 2A). The low yield may also be due to the preservation of the swab in silica beads and/or the lower density of buccal cells in the oral cavity of most bats. It is important to point out that despite the low comparative performance of buccal swabs, they still yielded enough DNA for PCR amplification. While buccal swabs may prove useful for larger bats, we recommend that further studies focus on testing a number of alternatives: 1) using cotton swabs rather than brushes, 2) using Whatman paper ‘brushes’ to see if buccal cells can be absorbed and preserved on paper better than with nylon bristles, and 3) combining the buccal swabs with preservation in a weakly acid lysis buffer that would lyse cells but preserve nuclei [35].

When comparing preservation media for wing punches, our results conclusively show that wing punches desiccated in silica beads in the field yield significantly greater quantities of DNA compared to samples preserved in ethanol or DMSO. Wing punches are an ideal tissue type for nonlethal bat sampling because they allow researchers to release the bat after capture. Punches are usually collected with a sterile biopsy puncher that standardizes the size of each sample independently of the collector’s skill, and can be easily stored in vials without further handling. Previous studies have shown that wounds from similarly-sized wing punches will heal completely in an average of 2–4 weeks [36].

Silica beads are affordable, easy to store, easy to acquire, and lack travel restrictions because they are not liquid or combustible. Compared to silica beads, ethanol and DMSO are inferior storage agents if the goal is to maximize DNA yield. These chemicals are also inherently damaging to DNA. Ethanol becomes increasingly acidic over time, breaking the sugar-phosphate backbone of DNA, and forming Maillard products—broken down sugars that may inhibit PCR [12,13,37]. We suggest that future studies test alternative storage media such as ethanol mixed with acid gelling agents to prevent acidification and DNA degradation over time. DMSO is both cytotoxic and genotoxic, and is known to lower base-pair composition dependence [38]. This is a useful feature to facilitate PCR amplification of, for example, a GC-rich sample [39,40]. At high concentration and for a period of years, DMSO buffer becomes deleterious and may cause PCR amplification to fail.

## CONCLUSION

We introduced mouth swabbing for collecting bat DNA, and tested techniques of preservation of tissues commonly used in the field. We found that brush-style mouth swabs did not yield as much nucleic acid concentration and distressed the bats more than collecting wing punches, and that storing wing punches in silica beads in the field yielded more DNA of higher quality compared with liquid buffers (ethanol and DMSO). We therefore recommend the use of silica beads as a field preservation medium for wing punches.

## MATERIALS AND METHODS

### Samples

Tissues were collected during field trips to Puerto Rico and Hispaniola (Dominican Republic) and represent nine species from two families: Mormoopidae (*Mormoops blainvillei*, *Pteronotus parnellii*, and *P*. *quadridens*) and Phyllostomidae (*Brachyphylla cavernarum*, *B*. *nana*, *Erophylla sezekorni*, *Macrotus waterhousii*, and *Monophyllus redmani*, see S1 Table). The sample collection protocols reported here were approved by Stony Brook University IACUC 20091741, Grand Valley State University IACUC 09-07-A, and California State University at Sacramento IACUC S10-001. Field work, sample collection and export from the Dominican Republic was approved by Ministry of the Environment permit no. 0000986.

For the sample type experiment, individuals caught on Mona Island and Puerto Rico in June 2010 were sampled by both brushing their cheeks and palates using buccal swab brushes and taking 2-mm diameter wing punches. Both brushes and wing punches from this trip were preserved dry in individual tubes and stored dry in a cabinet with permeable boxes of indicator silica beads to reduce humidity and prevent condensation. These samples were extracted shortly after arrival in the lab in 2010.

For the preservation medium experiment we sampled three 2-mm diameter wing punches from each captured individual during our first field collection to Puerto Rico and the Dominican Republic in May-June 2009. One punch per individual was preserved in the field dry in 3-mm indicating silica beads, in NaCl-saturated 20% DMSO, and in ethanol. Upon arrival at the lab the punches were frozen at −80°C and preserved ultra-cold until genomic DNA extraction in January 2011.

Wing tissues for both sample type and preservation medium experiments were obtained using Acu-Punch sterile, disposable 2-mm skin biopsy punches (Acuderm, Inc.). One biopsy punch was used for each individual, and skin samples were taken from regions of the plagiopatagium that were neither likely to tear into the edge of the wing, nor close to major blood vessels. Bats regularly make tears like these while flying into branches, and similar wounds have been shown to heal completely in less than 30 days [36]. Punches were preserved in ~0.7 g indicator silica, in ~1 mL of 20% DMSO solution saturated with NaCl, or in ~1 mL molecular grade 100% ethanol.

For the sample type experiment, Epicentre (Illumina, Inc.) brushes were used to swab the mouth of the bat for 60 s, minimizing bleeding or gagging. A timer was used to ensure that brushes were equally applied to all individuals. This procedure is standard for collecting samples from humans and other large mammals. The mouths of bats, however, do not open in a way that generates large cheek regions. For this reason, the brushes mostly sampled cells from the palate and the tongue.

### Extraction protocols

All DNA extractions were conducted in a laboratory that undergoes regular decontamination with hypochloride treatment. Each sample extraction was conducted separately to prevent cross contamination. All extractions were performed in a Bio-Safety Laboratory (BSL)-II cabinet, which was UV-irradiated for 1 hour prior to each sample extraction. All consumables, including pipettor tips, micro-centrifuge tubes, and collection tubes as well as the small equipment, such as pipettors, were UV-irradiated in a UV crosslinker for 20 minutes at 1200 x100μJ/cm^2^. Gloves were also changed between every step of the extraction to prevent contamination. Mock DNA extractions and control blank PCRs are performed continuously in the laboratory and screened for contamination.

Wing punches from both preservation medium and sample type experiments were placed in individual 1.5 μl micro-centrifuge tubes, and DNA was extracted using the QIAmp (Qiagen) tissue extraction protocol. First, all wing punches were lysed in 15 μl of ATL buffer with 10 μl of proteinase K and incubated at 56°C for 3 hours. Second, we added 50 μl of AL buffer with 1μl of carrier RNA and 50 μl of molecular-grade 99% ethanol, and incubated the solution at room temperature for 5 min. Third, the solution was purified using the QIAmp micro-columns. Finally, the samples were eluted using 55 μl of PCR-grade water and stored at 4°C prior to quantification and amplification.

Swab brushes from the sample type experiment were placed in 1.5 μl micro-centrifuge tubes, and DNA was extracted using a modified QIAmp extraction protocol. Cells from the swab brushes were lysed at 56°C for 60 min in 600 μl of QIAmp micro kit ATL buffer and 20 μl of proteinase K. Six hundred μl of AL buffer were added, with 1 μl of carrier RNA, and incubated the solution at 70°C for 10 min in a thermomixer shaking at 900 rpm. Three hundred μl of molecular-grade ethanol were added, and pulse-vortexed for each sample for 15 sec. All swab brushes were removed from the micro-centrifuge tubes, and the solution was purified using the QIAmp micro-columns. Finally, each sample was eluted using 55 μl of PCR-grade water and stored at 4°C prior to quantification and amplification.

### Measurement of nucleic acids and DNA yield

We expected that both the wing punches and buccal swabs would yield some DNA from non-chiropteran sources such as bacteria, fungi, and insects. Therefore, we used two measures of DNA yield: 1) total nucleic acid concentration using a spectrophotometer (Nanodrop 1000, Thermo Scientific), and 2) number of copies of a single-copy nuclear gene using quantitative PCR (qPCR). One and a half μL of DNA extract was used to obtain measures of absorbance at the 260-nm wavelength to determine optical density using the spectrophotometer with a sensitivity range from 2.0 to 3700 ng. Optical density measures can comprise RNA, protein, and chemical contamination in addition to DNA. We used the minimum threshold of 1.80 for the 260/280 optical density ratio to check for protein and chemical contamination [34]. Higher values indicate pure nucleic acids. For this reason we refer to optical density measures as nucleic acid quantifications, as they may include some RNA.

To specifically quantify target nuclear DNA rather than total nucleic acids or possible contaminants, we used a 124-bp fragment of the single-copy nuclear recombination-activating gene 2 (*rag2*) as the target locus for qPCR. The primers used for the protocol were designed based on chiropteran sequences of *rag2* obtained in our laboratory as well as those taken from Genbank: rag2-q2-f1 (5’-ACACCAAACAATGAGCTTTC-3’) and rag2-q2-r1 (5’-CCATATCTGGCTTCAGG-3’). Following primer design, a small subset of samples (*N* = 5) was used to verify the length of the target product and absence of mispriming using standard PCR. The PCR yielded a single low-molecular weight bright band of the correct length (S3 Fig.).

The Qiagen Quantifast SYBR green PCR kit was used to perform these reactions. The qPCR reactions followed the Qiagen SYBR Green PCR kit handbook, as follows: 12.5 μl of qPCR mix, 2.5 μl of nuclease free H_2_O, 2.5 μl of each primer, and 5 μl of the template for a total reaction volume of 25 μl. The conditions were a two-step cycling protocol with a 5-min PCR activation step at 95°C, denaturation for 10 min at 95°C and combined annealing and extension steps at 60°C for 30 s for a total number of 35 cycles. Quantitative PCR for each sample was replicated within runs (technical replicate), and in separate runs, resulting in ~4 estimates of gene copies per sample. The standard quantification curve used to estimate the number of copies in the sample was plotted using lambda bacteriophage DNA diluted from 10^8^ to 10^1^ concentration from a stock solution of 100 μg/ml or 10^10^ copies/ml. This curve was used to calculate the total number of DNA copies in the sample.

### Statistical analyses

After testing for normality within the sample type experiment and its treatments, a *t*-test was used to compare nucleic acid concentration from buccal cells and wing punches. Nucleic acid concentration from the preservation medium experiment was compared using a randomized block analysis of variance (ANOVA) with individuals as blocks and preservation methods as treatments.

Quantitative PCR depends strongly and nonlinearly on initial conditions, and requires replication of each DNA sample tested [41,42]. This introduces a multi-level structure to the data: observations of gene copy count are nested within qPCR runs as well as within the samples analyzed. Additionally, individual bats may vary in their behavior (when swabbing with the brush) or skin thickness (when taking a wing punch), introducing another source of variation. A hierarchical modeling approach was therefore used to measure the effects of preservation medium or sample type on single-copy gene quantity. The factors of interest for each experiment were either the sample type or preservation medium, and their effects were estimated using experiment-wide coefficients [43]. The variation arising from other factors, such as the qPCR run or identity of each bat were modeled as group-specific intercepts. The intercepts for individuals, in particular, were expected to capture variability in number of copies arising from bat species, condition, and time elapsed between collecting the punch in the field and extracting the DNA (as some bats sampled at the beginning of the field trip spent more time in field conditions than those captured closer to the date of return to the lab).

Multi-level models were fitted using maximum likelihood in the *lmer* function of the R package lme4 v. 0.999999-0 [44]. The fits of nested models to the data were compared using likelihood ratio tests, and significance of the log-likelihood ratio statistic was approximated by the *χ* ^2^ distribution with degrees of freedom equal to the difference in number of parameters of the models. For non-nested models, fits were compared using the Akaike Information Criterion (AIC), with the best-fit model corresponding to the lowest AIC value [45].

Analyses of correlation between DNA concentration and the number of copies detected were conducted by estimating Pearson’s *r* and its significance. To reduce the impact of extreme values in estimating correlations, up to four repeated measurements of gene copies per extract were summarized using the median. All analyses were carried out in R statistical language v. 2.14.2 [46].

## Supporting Information

S1 Fig Number of copies of single-copy nuclear gene obtained through quantitative PCR (qPCR) amplifications from different sample types and methods of preservation. Results are presented sorted by individual sampled. The models that best fit both datasets accounted for group-level effects of runs and individuals sampled (Table 1). A. Comparison between sample types obtained from the same individuals (no significant effect of sample type was found, see Table 1, note that individual WCL025 is not shown here). B. Comparison between preservation treatments for wing punch samples obtained from the same individuals (ethanol and dry preservation were significantly better than DMSO preservation, see Table 1, note that individual ALR074 is not shown here).
(TIFF)

S2 Fig Relationship between the median number of copies of the single-copy nuclear gene obtained from repeated quantitative PCR reactions and concentration of whole DNA (buccal cells and wing punches). The correlation was not significant (*r* = 0.07, *p* = 0.766).
(TIFF)

S3 Fig Standard PCR verification of *rag2* quantitative PCR primers used in the study. Lane 1 is a 100-bp ladder, lanes 2–4 are sample WCL025, lanes 5–7 are sample ALR011, lanes 8–10 are negative controls. Lanes 1 and 5 used primer pair rag2-q2-f1/ rag2-q2-r1 (included in study), lanes 2 and 6 used primers rag2-q2-f2/ rag2-q2-r2 (not included), lanes 3 and 7 used primers rag2-q2-f3/ rag2-q2-r3 (not included). This image was modified from the original by inverting black and white and showing only the top lanes. No other modifications were performed to the image.
(TIFF)

S1 Table List of samples, sample types, and preservation media used in the two experiments. DMSO is NaCl-saturated dimethyl sulfoxide, ETOH is ethanol, and SG is silica desiccant.
(DOCX)
