# Supplementary figures and images for: From the Field to the Lab: Best Practices for Field Preservation of Bat Specimens for Molecular Analyses
Source: PLoS One. 2015 Mar 23;10(3):e0118994. doi: 10.1371/journal.pone.0118994 (PMC4370412; doi:10.1371/journal.pone.0118994)

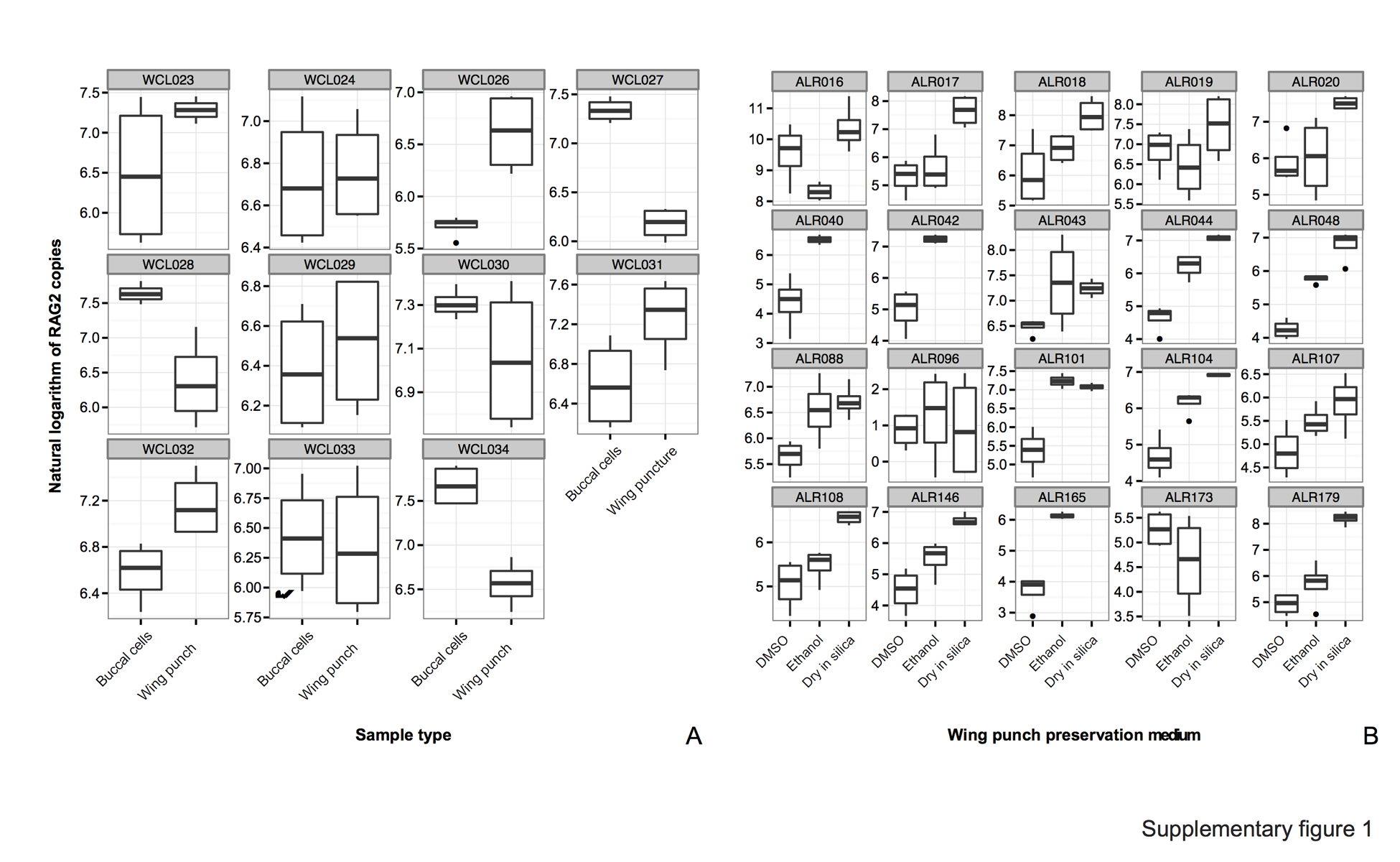

Supplement: S1 Fig — Results are presented sorted by individual sampled. The models that best fit both datasets accounted for group-level effects of runs and individuals sampled (Table 1). A. Comparison between sample types obtained from the same individuals (no significant effect of sample type was found, see Table 1, note that individual WCL025 is not shown here). B. Comparison between preservation treatments for wing punch samples obtained from the same individuals (ethanol and dry preservation were significantly better than DMSO preservation, see Table 1, note that individual ALR074 is not shown here). (TIFF) [file pone.0118994.s001.tiff]

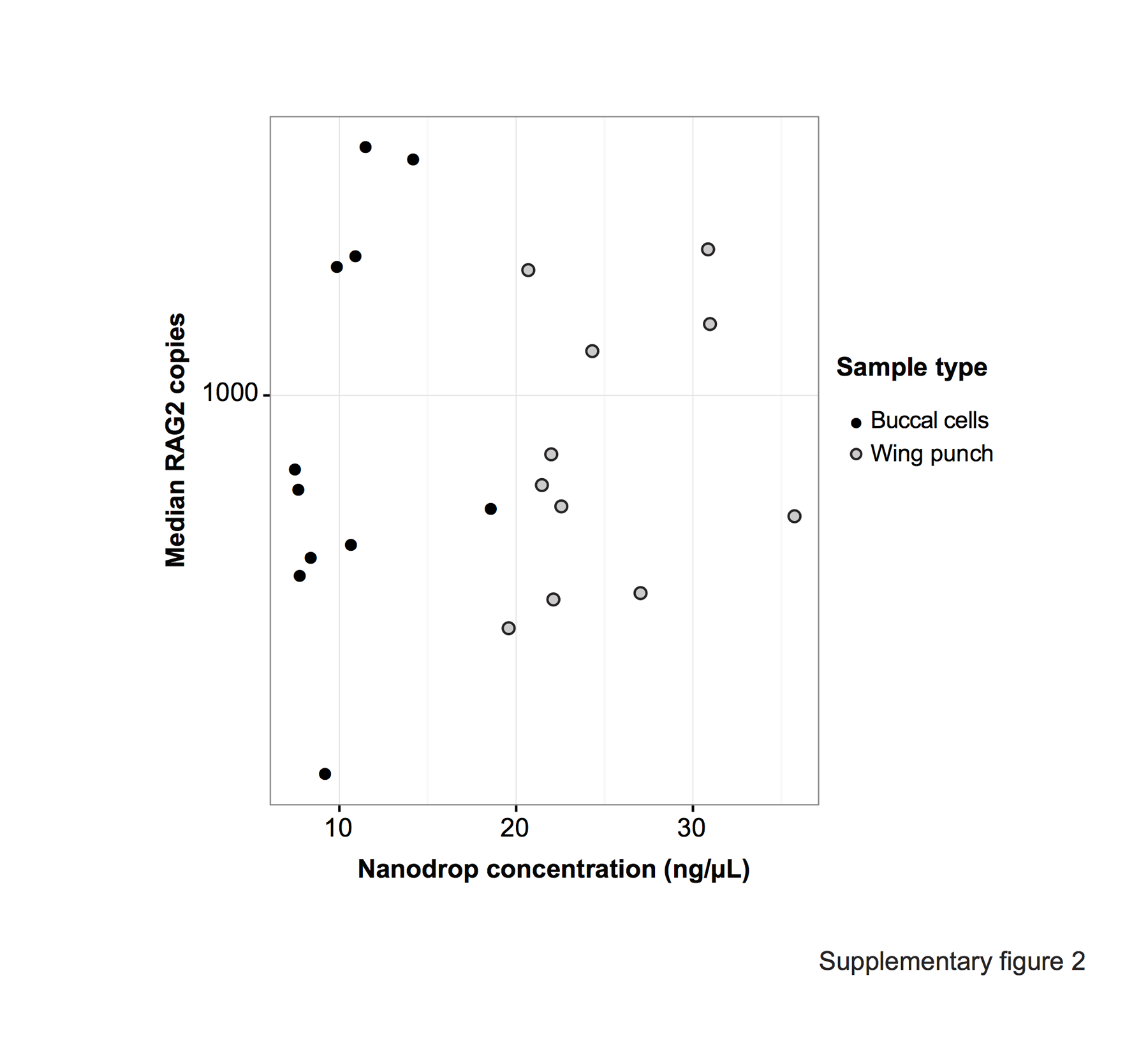

Supplement: S2 Fig — The correlation was not significant (r = 0.07, p = 0.766). (TIFF) [file pone.0118994.s002.tiff]

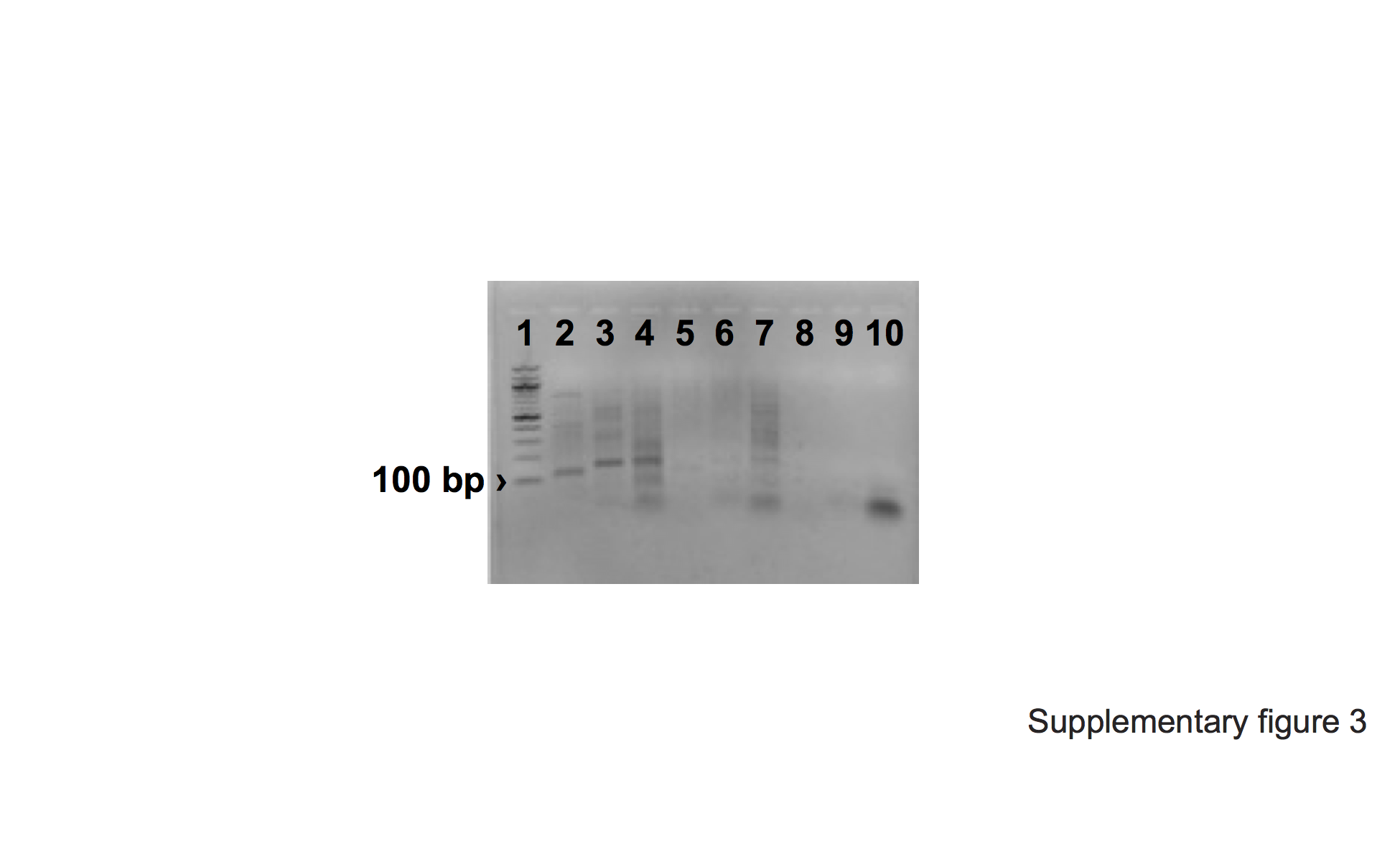

Supplement: S3 Fig — Lane 1 is a 100-bp ladder, lanes 2–4 are sample WCL025, lanes 5–7 are sample ALR011, lanes 8–10 are negative controls. Lanes 1 and 5 used primer pair rag2-q2-f1/ rag2-q2-r1 (included in study), lanes 2 and 6 used primers rag2-q2-f2/ rag2-q2-r2 (not included), lanes 3 and 7 used primers rag2-q2-f3/ rag2-q2-r3 (not included). This image was modified from the original by inverting black and white and showing only the top lanes. No other modifications were performed to the image. (TIFF) [file pone.0118994.s003.tiff]
