# Supplementary material for: From the Field to the Lab: Best Practices for Field Preservation of Bat Specimens for Molecular Analyses
Source: PLoS One. 2015 Mar 23;10(3):e0118994. doi: 10.1371/journal.pone.0118994 (PMC4370412; doi:10.1371/journal.pone.0118994)
Supplement: S1 Table — DMSO is NaCl-saturated dimethyl sulfoxide, ETOH is ethanol, and SG is silica desiccant. (DOCX) [file pone.0118994.s004.docx]

**Supporting Table 1**. List of samples, sample types, and preservation media used in the two experiments. DMSO is NaCl-saturated dimethyl sulfoxide, ETOH is ethanol, and SG is silica desiccant.

| Sample ID | Sample Type | Medium | Experiment | Genus | Species | Country | Collection Date |
| --- | --- | --- | --- | --- | --- | --- | --- |
| ALR016 | wing puncture | DMSO | Preservation medium | *Brachyphylla* | *cavernarum* | Puerto Rico | 28-May-2009 |
| ALR016 | wing puncture | ETOH | Preservation medium | *Brachyphylla* | *cavernarum* | Puerto Rico | 28-May-2009 |
| ALR016 | wing puncture | SG | Preservation medium | *Brachyphylla* | *cavernarum* | Puerto Rico | 28-May-2009 |
| ALR017 | wing puncture | DMSO | Preservation medium | *Mormoops* | *blainvillei* | Puerto Rico | 28-May-2009 |
| ALR017 | wing puncture | ETOH | Preservation medium | *Mormoops* | *blainvillei* | Puerto Rico | 28-May-2009 |
| ALR017 | wing puncture | SG | Preservation medium | *Mormoops* | *blainvillei* | Puerto Rico | 28-May-2009 |
| ALR018 | wing puncture | DMSO | Preservation medium | *Mormoops* | *blainvillei* | Puerto Rico | 28-May-2009 |
| ALR018 | wing puncture | ETOH | Preservation medium | *Mormoops* | *blainvillei* | Puerto Rico | 28-May-2009 |
| ALR018 | wing puncture | SG | Preservation medium | *Mormoops* | *blainvillei* | Puerto Rico | 28-May-2009 |
| ALR019 | wing puncture | DMSO | Preservation medium | *Monophyllus* | *redmani* | Puerto Rico | 28-May-2009 |
| ALR019 | wing puncture | ETOH | Preservation medium | *Monophyllus* | *redmani* | Puerto Rico | 28-May-2009 |
| ALR019 | wing puncture | SG | Preservation medium | *Monophyllus* | *redmani* | Puerto Rico | 28-May-2009 |
| ALR020 | wing puncture | DMSO | Preservation medium | *Monophyllus* | *redmani* | Puerto Rico | 28-May-2009 |
| ALR020 | wing puncture | ETOH | Preservation medium | *Monophyllus* | *redmani* | Puerto Rico | 28-May-2009 |
| ALR020 | wing puncture | SG | Preservation medium | *Monophyllus* | *redmani* | Puerto Rico | 28-May-2009 |
| ALR040 | wing puncture | DMSO | Preservation medium | *Pteronotus* | *parnellii* | Puerto Rico | 29-May-2009 |
| ALR040 | wing puncture | ETOH | Preservation medium | *Pteronotus* | *parnellii* | Puerto Rico | 29-May-2009 |
| ALR042 | wing puncture | DMSO | Preservation medium | *Pteronotus* | *parnellii* | Puerto Rico | 31-May-2009 |
| ALR042 | wing puncture | ETOH | Preservation medium | *Pteronotus* | *parnellii* | Puerto Rico | 31-May-2009 |
| ALR043 | wing puncture | DMSO | Preservation medium | *Pteronotus* | *parnellii* | Puerto Rico | 31-May-2009 |
| ALR043 | wing puncture | ETOH | Preservation medium | *Pteronotus* | *parnellii* | Puerto Rico | 31-May-2009 |
| ALR043 | wing puncture | SG | Preservation medium | *Pteronotus* | *parnellii* | Puerto Rico | 31-May-2009 |
| ALR044 | wing puncture | DMSO | Preservation medium | *Pteronotus* | *quadridens* | Puerto Rico | 31-May-2009 |
| ALR044 | wing puncture | ETOH | Preservation medium | *Pteronotus* | *quadridens* | Puerto Rico | 31-May-2009 |
| ALR044 | wing puncture | SG | Preservation medium | *Pteronotus* | *quadridens* | Puerto Rico | 31-May-2009 |
| ALR048 | wing puncture | DMSO | Preservation medium | *Mormoops* | *blainvillei* | Puerto Rico | 31-May-2009 |
| ALR048 | wing puncture | ETOH | Preservation medium | *Mormoops* | *blainvillei* | Puerto Rico | 31-May-2009 |
| ALR048 | wing puncture | SG | Preservation medium | *Mormoops* | *blainvillei* | Puerto Rico | 31-May-2009 |
| ALR074 | wing puncture | ETOH | Preservation medium | *Brachyphylla* | *cavernarum* | Puerto Rico | 2-Jun-2009 |
| ALR088 | wing puncture | DMSO | Preservation medium | *Pteronotus* | *quadridens* | Puerto Rico | 2-Jun-2009 |
| ALR088 | wing puncture | ETOH | Preservation medium | *Pteronotus* | *quadridens* | Puerto Rico | 2-Jun-2009 |
| ALR088 | wing puncture | SG | Preservation medium | *Pteronotus* | *quadridens* | Puerto Rico | 2-Jun-2009 |
| ALR096 | wing puncture | DMSO | Preservation medium | *Macrotus* | *waterhousii* | Dominican Republic | 7-Jun-2009 |
| ALR096 | wing puncture | ETOH | Preservation medium | *Macrotus* | *waterhousii* | Dominican Republic | 7-Jun-2009 |
| ALR096 | wing puncture | SG | Preservation medium | *Macrotus* | *waterhousii* | Dominican Republic | 7-Jun-2009 |
| ALR101 | wing puncture | DMSO | Preservation medium | *Brachyphylla* | *pumila* | Dominican Republic | 7-Jun-2009 |
| ALR101 | wing puncture | ETOH | Preservation medium | *Brachyphylla* | *pumila* | Dominican Republic | 7-Jun-2009 |
| ALR101 | wing puncture | SG | Preservation medium | *Brachyphylla* | *pumila* | Dominican Republic | 7-Jun-2009 |
| ALR104 | wing puncture | DMSO | Preservation medium | *Erophylla* | *sezekorni* | Dominican Republic | 8-Jun-2009 |
| ALR104 | wing puncture | ETOH | Preservation medium | *Erophylla* | *sezekorni* | Dominican Republic | 8-Jun-2009 |
| ALR104 | wing puncture | SG | Preservation medium | *Erophylla* | *sezekorni* | Dominican Republic | 8-Jun-2009 |
| ALR107 | wing puncture | DMSO | Preservation medium | *Erophylla* | *sezekorni* | Dominican Republic | 8-Jun-2009 |
| ALR107 | wing puncture | ETOH | Preservation medium | *Erophylla* | *sezekorni* | Dominican Republic | 8-Jun-2009 |
| ALR107 | wing puncture | SG | Preservation medium | *Erophylla* | *sezekorni* | Dominican Republic | 8-Jun-2009 |
| ALR108 | wing puncture | DMSO | Preservation medium | *Pteronotus* | *parnellii* | Dominican Republic | 9-Jun-2009 |
| ALR108 | wing puncture | ETOH | Preservation medium | *Pteronotus* | *parnellii* | Dominican Republic | 9-Jun-2009 |
| ALR108 | wing puncture | SG | Preservation medium | *Pteronotus* | *parnellii* | Dominican Republic | 9-Jun-2009 |
| ALR146 | wing puncture | DMSO | Preservation medium | *Pteronotus* | *quadridens* | Dominican Republic | 11-Jun-2009 |
| ALR146 | wing puncture | ETOH | Preservation medium | *Pteronotus* | *quadridens* | Dominican Republic | 11-Jun-2009 |
| ALR146 | wing puncture | SG | Preservation medium | *Pteronotus* | *quadridens* | Dominican Republic | 11-Jun-2009 |
| ALR165 | wing puncture | DMSO | Preservation medium | *Pteronotus* | *parnellii* | Dominican Republic | 13-Jun-2009 |
| ALR165 | wing puncture | ETOH | Preservation medium | *Pteronotus* | *parnellii* | Dominican Republic | 13-Jun-2009 |
| ALR173 | wing puncture | DMSO | Preservation medium | *Monophyllus* | *redmani* | Dominican Republic | 13-Jun-2009 |
| ALR173 | wing puncture | ETOH | Preservation medium | *Monophyllus* | *redmani* | Dominican Republic | 13-Jun-2009 |
| ALR179 | wing puncture | DMSO | Preservation medium | *Brachyphylla* | *pumila* | Dominican Republic | 13-Jun-2009 |
| ALR179 | wing puncture | ETOH | Preservation medium | *Brachyphylla* | *pumila* | Dominican Republic | 13-Jun-2009 |
| ALR179 | wing puncture | SG | Preservation medium | *Brachyphylla* | *pumila* | Dominican Republic | 13-Jun-2009 |
| WCL023 | buccal swab | dry | Sample type | *Monophyllus* | *redmani* | Puerto Rico | 4-Jun-2010 |
| WCL023 | wing puncture | SG | Sample type | *Monophyllus* | *redmani* | Puerto Rico | 4-Jun-2010 |
| WCL024 | buccal swab | dry | Sample type | *Monophyllus* | *redmani* | Puerto Rico | 4-Jun-2010 |
| WCL024 | wing puncture | SG | Sample type | *Monophyllus* | *redmani* | Puerto Rico | 4-Jun-2010 |
| WCL025 | wing puncture | SG | Sample type | *Monophyllus* | *redmani* | Puerto Rico | 4-Jun-2010 |
| WCL026 | buccal swab | dry | Sample type | *Monophyllus* | *redmani* | Puerto Rico | 4-Jun-2010 |
| WCL026 | wing puncture | SG | Sample type | *Monophyllus* | *redmani* | Puerto Rico | 4-Jun-2010 |
| WCL027 | buccal swab | dry | Sample type | *Monophyllus* | *redmani* | Puerto Rico | 4-Jun-2010 |
| WCL027 | wing puncture | SG | Sample type | *Monophyllus* | *redmani* | Puerto Rico | 4-Jun-2010 |
| WCL028 | buccal swab | dry | Sample type | *Monophyllus* | *redmani* | Puerto Rico | 4-Jun-2010 |
| WCL028 | wing puncture | SG | Sample type | *Monophyllus* | *redmani* | Puerto Rico | 4-Jun-2010 |
| WCL029 | buccal swab | dry | Sample type | *Monophyllus* | *redmani* | Puerto Rico | 4-Jun-2010 |
| WCL029 | wing puncture | SG | Sample type | *Monophyllus* | *redmani* | Puerto Rico | 4-Jun-2010 |
| WCL030 | buccal swab | dry | Sample type | *Artibeus* | *jamaicensis* | Puerto Rico | 6-Jun-2010 |
| WCL030 | wing puncture | SG | Sample type | *Artibeus* | *jamaicensis* | Puerto Rico | 6-Jun-2010 |
| WCL031 | buccal swab | dry | Sample type | *Artibeus* | *jamaicensis* | Puerto Rico | 6-Jun-2010 |
| WCL031 | wing puncture | SG | Sample type | *Artibeus* | *jamaicensis* | Puerto Rico | 6-Jun-2010 |
| WCL032 | buccal swab | dry | Sample type | *Artibeus* | *jamaicensis* | Puerto Rico | 6-Jun-2010 |
| WCL032 | wing puncture | SG | Sample type | *Artibeus* | *jamaicensis* | Puerto Rico | 6-Jun-2010 |
| WCL033 | buccal swab | dry | Sample type | *Artibeus* | *jamaicensis* | Puerto Rico | 6-Jun-2010 |
| WCL033 | wing puncture | SG | Sample type | *Artibeus* | *jamaicensis* | Puerto Rico | 6-Jun-2010 |
| WCL034 | buccal swab | dry | Sample type | *Artibeus* | *jamaicensis* | Puerto Rico | 7-Jun-2010 |
| WCL034 | wing puncture | SG | Sample type | *Artibeus* | *jamaicensis* | Puerto Rico | 7-Jun-2010 |
